# Mitochondrial genome plasticity of mammalian species

**DOI:** 10.1186/s12864-024-10201-9

**Published:** 2024-03-14

**Authors:** Bálint Biró, Zoltán Gál, Zsófia Fekete, Eszter Klecska, Orsolya Ivett Hoffmann

**Affiliations:** 1 Agribiotechnology and Precision Breeding for Food Security National Laboratory, Department of Animal Biotechnology, Institute of Genetics and Biotechnology, Hungarian University of Agriculture and Life Sciences, Szent-Györgyi Albert str. 4, 2100 Gödöllő, Hungary; 2 Group BM, Data Insights Team, _VOIS, Kerepesi str. 35, 1087 Budapest, Hungary; 3 Department of Genetics and Genomics, Institute of Genetics and Biotechnology, Hungarian University of Agriculture and Life Sciences, Szent-Györgyi Albert str. 4, 2100 Gödöllő, Hungary; 4 FamiCord Group, Krio Institute, Kelemen László str, 1026 Budapest, Hungary

**Keywords:** NUMT, Mammals, Genome, Bioinformatics, Machine learning

## Abstract

There is an ongoing process in which mitochondrial sequences are being integrated into the nuclear genome. The importance of these sequences has already been revealed in cancer biology, forensic, phylogenetic studies and in the evolution of the eukaryotic genetic information. Human and numerous model organisms’ genomes were described from those sequences point of view. Furthermore, recent studies were published on the patterns of these nuclear localised mitochondrial sequences in different taxa.

However, the results of the previously released studies are difficult to compare due to the lack of standardised methods and/or using few numbers of genomes. Therefore, in this paper our primary goal is to establish a uniform mining pipeline to explore these nuclear localised mitochondrial sequences.

Our results show that the frequency of several repetitive elements is higher in the flanking regions of these sequences than expected. A machine learning model reveals that the flanking regions’ repetitive elements and different structural characteristics are highly influential during the integration process.

In this paper, we introduce a general mining pipeline for all mammalian genomes. The workflow is publicly available and is believed to serve as a validated baseline for future research in this field. We confirm the widespread opinion, on - as to our current knowledge - the largest dataset, that structural circumstances and events corresponding to repetitive elements are highly significant. An accurate model has also been trained to predict these sequences and their corresponding flanking regions.

**Supplementary Information:**

The online version contains supplementary material available at 10.1186/s12864-024-10201-9.

## Introduction

At the beginning of the evolution of multicellular organisms, an intracellular cooperation occurred between alpha-proteobacteria and Archaea [1, 2]. One of the organelles that have formed during this cooperation is the mitochondria, which is primarily responsible for oxidative phosphorylation, however it also participates in several other intracellular processes [2]. One of the unique phenotypic characteristics of the mitochondria is that it has its own genome (mtDNA), which is considered as the most important evidence for the endosymbiotic theory [1]. During the evolution of eukaryotes, endosymbiotic gene transfer (EGT) occurred, resulting in a large amount of genetic material being transferred from the organellar genomes to the host cell’s nuclear genome [3, 4]. The process in which mitochondrial sequences transfer to the nuclear genome is referred to as NUMTogenesis [5].

The integration of certain parts of organelle genomes into other genomes was first described in the case of the maize mitochondria and chloroplasts [6]. The presence of NUMTs in animals was first proven in the genome of the domestic cat. The NUMTs found in the cat genome are unique in several ways. On one hand, the cat nuclear genome contains almost half of the corresponding mtDNA, a 7.9 kb sequence. On the other hand, this large NUMT is present in a copy number that is several tens of times [7]. Larger NUMTs that cover almost the entire mtDNA (megaNUMTs) have also been identified in human samples [8]. Approximately 140 NUMTs have been described in the human genome [9]. NUMTs are also present in the genome of the honeybee, and their number is almost ten times higher than that of NUMTs found in the human genome [10]. The genome of organelles of endosymbiotic origin is usually less than 0.05% of their independent ancestors. Therefore, a significant part of the products of the host cell’s genome must be redirected to the mitochondria [11]. The molecular driving force behind EGT is explained by Müller’s theory [12]. According to this theory, deletions will occur in a genome that is sexually isolated, i.e. without recombination (in the case of NUMTogenesis, the mtDNA itself), which contributes to the loss of genetic material of the given genome. This results in the erosion of the genome in the short term and leading to the disappearance of the genome in the long term [13–15], mtDNA is sexually isolated because it is uniparentally inherited in most eukaryotes [16]. Therefore, the mtDNA is influenced by the effect defined by Müller [17], and the operation of EGT, by directing mitochondrial-derived genetic material into the cell nucleus, rescues the mtDNA from the degrading effect of Müller’s theory [18].

Another assumption is that maintaining multiple organelles per cell and multiple genomes per organelle requires too much energy investment from the cell. Therefore, the transfer of organelle genomes through EGT to the cell nucleus, and then redirecting the gene products back to the organelles is a less energy-intensive process than if each organelle had to do it on its own [11].

Environmental factors can lead to the transfer of genetic material from mitochondria to the nucleus, referred to as NUMTogens [5]. NUMTogens are physical [19], chemical [20] and biological stresses that increase the level of mitochondrial toxicity or stress, causing damage to the mitochondrial membrane which is the first step in the NUMTogenesis process. The disruption of the integrity of the mitochondrial membrane, resulting from events such as the excessive production of reactive oxygen species (ROS), the release of cytochrome c, and mitophagy, can allow mtDNA to escape from mitochondria. While the production of reactive oxygen species is generally considered as a random occurrence, the latter two take place during gametogenesis and are therefore largely controlled [5]. On top of that, the disruption of the integrity of the mitochondrial membrane can be caused by both external factors that induce mitostress (ionizing radiation, aging, endotoxins, ROS, endonucleases) and mutations [21]. These mitostressors damage the mtDNA to a greater extent than the nuclear genomic DNA (nDNAs) when exposed to radiation, probably due to the higher efficiency of the repair mechanisms in the cell nucleus [22]. The induction of NUMTogenesis by ionizing radiation has been proven in chicken embryos and yeast cells [19, 23]. In another study, the presence of NUMTs in the brain and liver tissue of rats was investigated over time [24]. Results showed that the number of NUMTs increased in older tissues. The increased frequency of NUMTs in aging cells has also been proven in yeast [25].

When the mitochondrial membrane is damaged, the organelle receives a degradation signal which induces the phenomenon of mitophagy. Mitophagy is a special case of autophagy that takes place in the mitophagosome [26]. This organelle is responsible for the degradation of damaged mitochondria and the recycling of its components [27]. In case of inadequate mitophagy, mitochondrial-originated sequences enter the cytoplasm. According to other theories, the entry of these sequences into the cytoplasm mainly occurs due to inadequate division and membrane fusion events [20, 27]. NUMT precursors are protected in the cytoplasm from the digestion of nucleases by a vesicle-mediated route or through the formation of a complex with histone-like proteins that bind to DNA. Mitochondrial-originated sequences located in the cytoplasm enter the nucleus through membrane fusion and/or pores [20]. After entering the nucleus, NUMT precursors can be incorporated into the nuclear genome during Double Stranded Breaks (DSB) through the non-homologous end joining (NHEJ) DNA repair mechanism. From the moment of incorporation, these sequences can be referred to as NUMTs. Mitostressors also increase the frequency of DSBs in nDNA [22]. In the case of NHEJ, a nuclease-mediated deletion always occurs in the absence of template DNA. NHEJ often results in a long single-stranded DNA segment, which increases the risk of longer deletions and translocations. According to some explanations, the cell uses NUMT precursors as template DNA to prevent larger damage caused by deletions and translocations [27].

Here we performed a systematic investigation of NUMTs across all the NCBI mammalian genomes.

We have performed this investigation in order to test the hypothesis whether all mammalian species indeed harbour NUMTs integrated within their nuclear genomes.

Based on this hypothesis our goal was also to explore the characteristics of the described NUMTs.

Furthermore, we aimed to identify the factors influencing NUMTogenesis. The motivation behind that particular hypothesis testing was to generalize our knowledge about this biological phenomenon. Previous studies have described NUMTogenesis in an isolated form with small sample sizes and different approaches, our investigation is intended to contribute to the field by providing comparable insights.

Our other hypothesis was that it is possible to predict NUMTs by utilizing modern machine learning based methods. This part is solely motivated by its practicality given that having NUMTs in an experimental setup (mainly during genome assembly and genome modification) often leads to biased, unreliable results [28–30]. Furthermore, by utilizing predictive techniques, one can eliminate the necessity of a complete reference genome when it comes to NUMT detection, hence there is no need to perform sequence alignment which is considered a computationally intensive process.

## Results

### Repetitive elements and NUMTs

Several repetitive elements were found that have different frequency in the 200 bp flanking regions of NUMTs than it should be expected. Three distinct behaviours could be observed when it comes to repetitive elements.

**Fig. 1:**
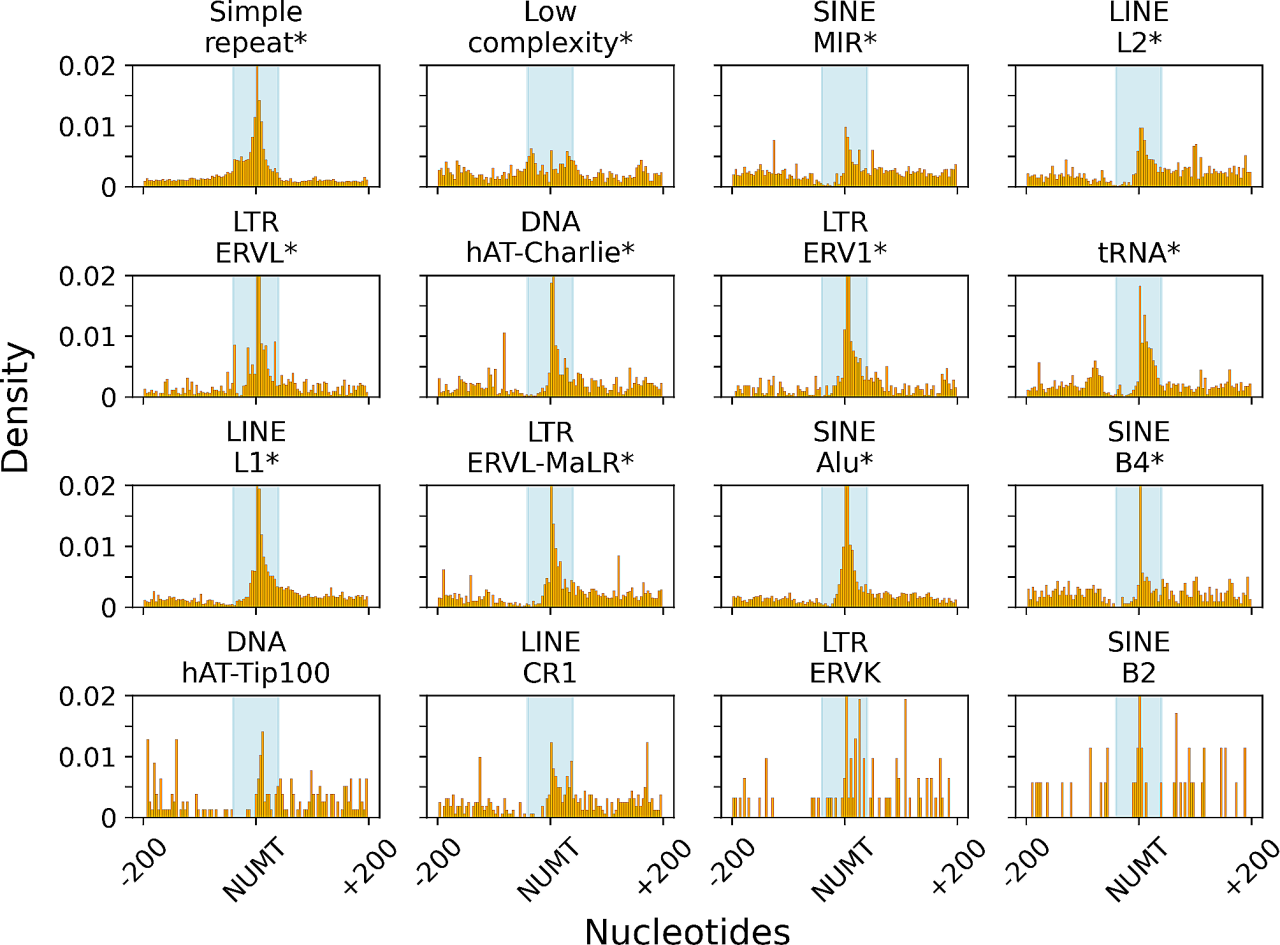
The frequency patterns of repetitive elements in the 200 bp flanking regions of NUMTs. Blue shaded areas are +-20 bps from NUMTs. Asterisk indicates *p* value < 0.05

Simple repeat, Long Terminal Repeat Endogenous RetroVirus-related eLement (LTR ERVL) and low complexity elements are getting more frequent in close proximity of the upstream flanking region of NUMTs. However, the frequency of those elements is going down in distant downstream flanking regions. In the above cases, the repetitive elements have the highest density in NUMTs (Fig. 1).

While as the second particular behaviour, the frequencies of Short Interspersed Nuclear Element Mammalian-wide Interspersed Repeats (SINE MIR), Long Interspersed Nuclear Element L2 (LINE L2), DNA hAT-Charlie, LTR ERV1, tRNA, LINE L1, LTR ERVL-MaLR, SINE Alu and SINE B4 elements drop drastically in close upstream proximity of NUMTs, the frequency of these elements are getting sparser in distant downstream flanking of NUMTs. These elements also have their corresponding peak frequency in NUMTs. The distributions of the repetitive elements mentioned above significantly differ from random distribution (*p* value < 0.05).

In the third behaviour, there is no pattern in the case of sparse repetitive elements (DNA hAT-Tip100, LINE CR1, LTR ERVK, SINE B2), hence the distribution of these elements does not differ from random distributions (*p* value < 0.05) (Fig. 1). However, several repetitive elements did not show a particular pattern in the close proximity of NUMTs hence only 4 of them are displayed in the last row of Fig. 1.

### Descriptive analysis of NUMTs

**Fig. 2:**
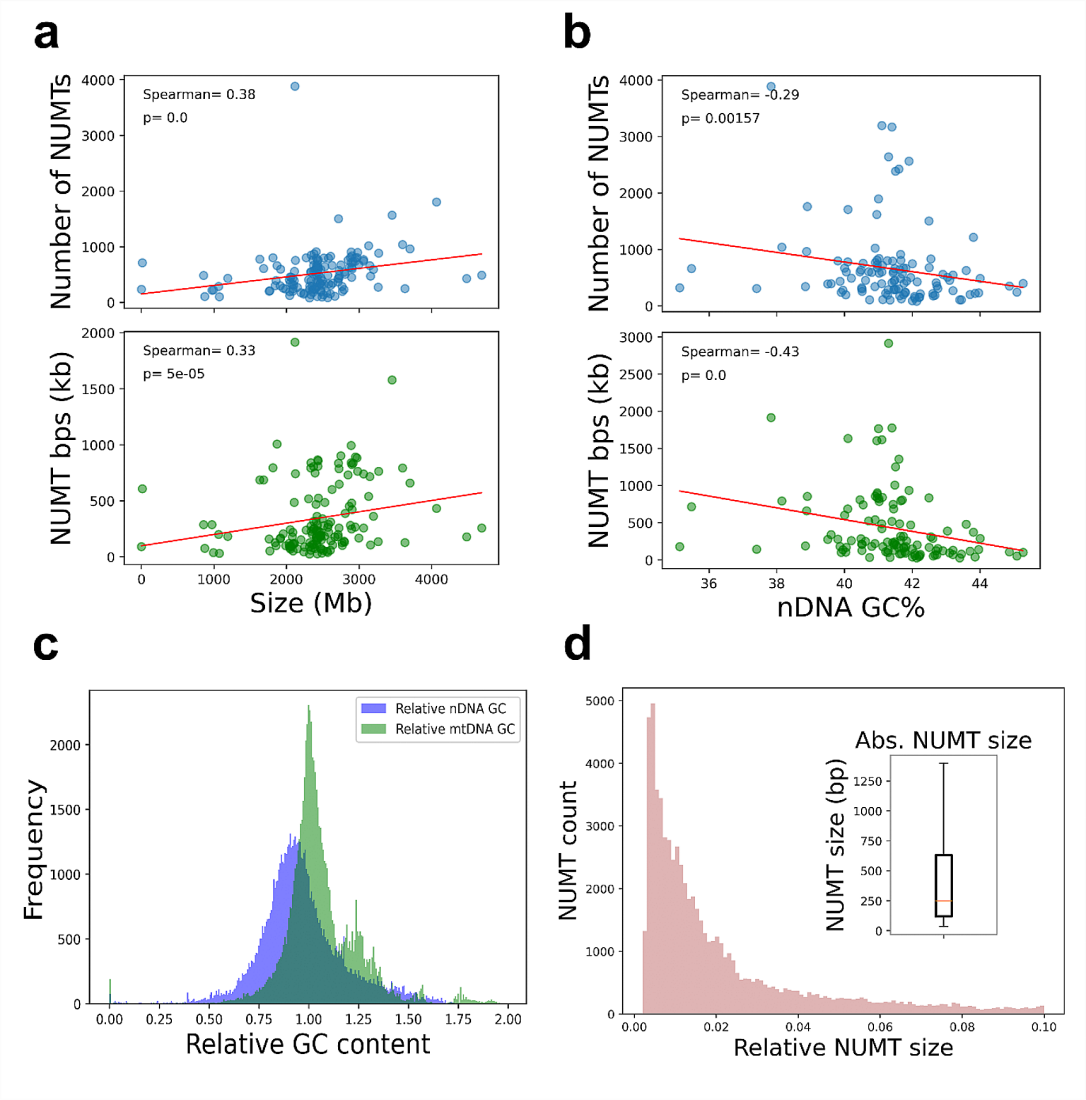
Descriptive statistics of the NCBI mammalian NUMTs. (**a**) Correlation between NUMT length/count and nuclear genome size in Mb. (**b**) Correlation between NUMT length/count and total nuclear genome GC content (%). (**c**) The relative GC contents of NUMTs and their corresponding mitochondrial sequences compared to the GC content of total nuclear genome and total mtDNA genome GC contents respectively. (**d**) Distributions of relative and absolute NUMT sizes. Relativization was performed based on the corresponding mtDNA genome size

Most of the analysed nuclear genomes were between 2000 and 3000 Mb in size and had 0.4–0.42 GC ratio. We found a moderated positive Spearman correlation between nuclear genome sizes and number/cumulative length of NUMTs (0.38 and 0.33 correlation coefficients respectively with *p* values < 0.01) (Fig. 2/a). This means that the larger the nuclear genome the more NUMTs will be inserted.

By contrast, we found that the GC content of the nuclear genome has an opposite effect. Namely, the higher the GC content, the smaller the number and cumulative length of NUMTs (-0.29 and − 0.43 Spearman correlation coefficient respectively with *p* values < 0.01) (Fig. 2/b).

NUMTs tend to have lower GC content than their host nuclear genome (lower mean than 1.0). Meanwhile their corresponding mitochondrial sequences have a 1.0 centered distribution (mean ∼ 1.0) compared to their parent mtDNA genome when it comes to GC content. However, a subpopulation of NUMTs with around 1.25 relative GC content can be observed (Fig. 2/c). Since the GC content of the mtDNA is usually lower than nDNA’s GC content, this result can be considered as a feature that strengthens the theory of the mitochondrial origin of NUMTs.

The distribution of relative NUMT sizes is highly skewed toward smaller NUMTs (Fig. 2/d). The absolute NUMT sizes can be described with a 121, 632 interquartile range and a 248 median. We found nine species (*Delphinapterus leucas, Tursiops truncatus, Castor canadensis, Cavia porcellus, Globicephala melas, Lagenorhynchus obliquidens, Monodelphis domestica, Orcinus orca, Theropithecus gelada* and *Tursiops truncatus*) with their total mtDNA genomes integrated into their nuclear genomes as NUMTs. In the *Castor canadensis* nuclear genome, two concatenated mtDNA genomes are present as a megaNUMT.

### Order specific patterns of NUMTogenesis

**Fig. 3:**
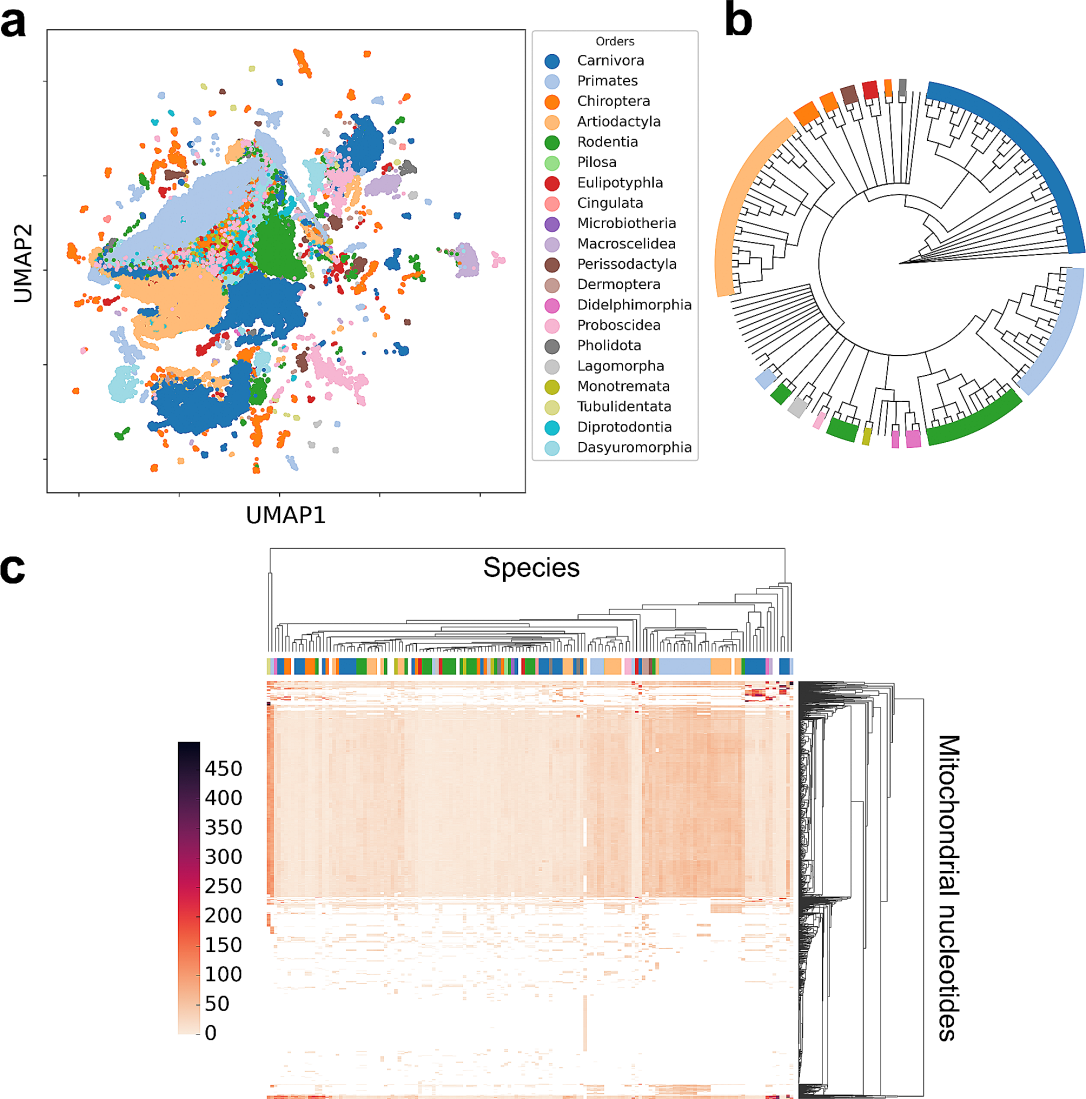
NUMT features integrated with taxonomical data. (**a**) UMAP dimension reduction of NUMT features coloured by taxonomical order. (**b**) Consensus phylogenetic tree with the General Time Reversible (GTR) model and 100 bootstrap iterations of the corresponding mammalian mtDNA genomes. (**c**) Heatmap shows how many times a given nucleotide contributed to NUMTogenesis. Columns represent species while rows represent mitochondrial nucleotides

The NUMTs of orders that have several genomes involved, are clustered together based on NAC, kmers, NMBroto, Z-curve and mismatch profile iFeatureOmegaCLI features. For example, Artiodactyla related NUMTs which are from 25 genomes are tightly clustered together. The same applies to Rodentia and Primates. By contrast, Didelphimorphia related NUMTs that are derived from only two genomes, are scatteredand do not form such distinct clusters (Fig. 3/a, b).

Primates is the only order that is clustered tightly together when it comes to the number of nucleotide involvement in the process of NUMTogenesis (Fig. 3/c).

In general, most of the mtDNA genome is involved in NUMTogenesis throughout mammalian species. However, position specific discrepancies can be observed. Namely that the ends of the mtDNA genomes show huge differences in terms of the NUMTogenesis involvement of particular nucleotides (Fig. 3/c).

### Machine learning approach for NUMT classification

**Fig. 4:**
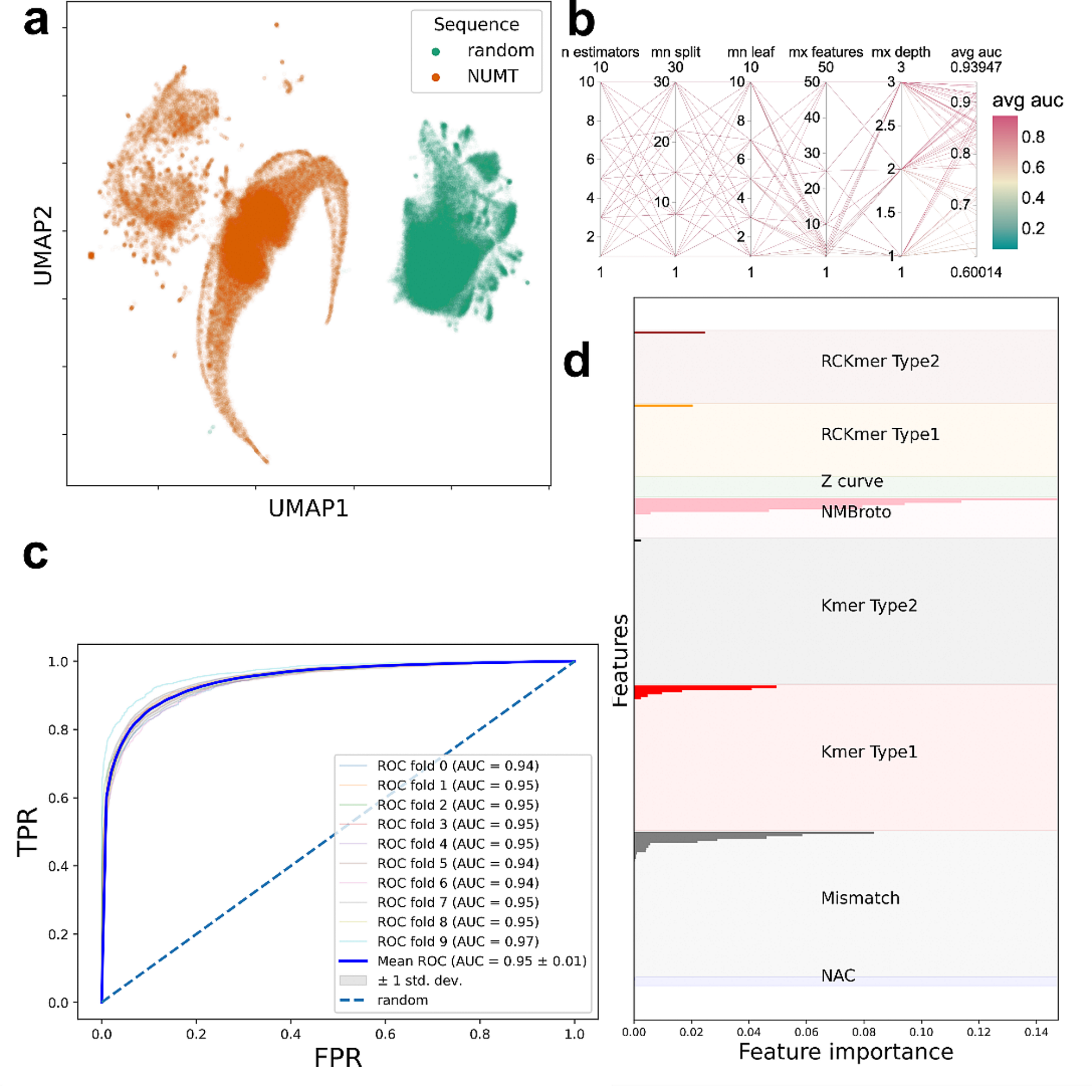
NUMT and random sequence classification with random forest algorithm. (**a**) UMAP dimension reduction of NUMT and random sequence features. (**b**) Parallel coordinates diagram of the hyperparameter optimisation. (**c**) k-fold cross validation ROC analysis. (**d**) Feature importances of iFeatureOmegaCLI’s features

As expected, NUMTs and random sequences form distinctly separated UMAP clusters based on the previously mentioned features despite the very few numbers of data points closer to the opposite cluster. However, NUMTs are divided into two subclusters (Fig. 4/a).

Nevertheless, no distinct pattern could be detected between these subclusters in terms of taxonomical order i.e. order specific UMAP points did not form distinct clusters.

Random search hyperparameter optimisation of a random forest model resulted in a maximum AUROC of 0.94. During the classification, 7 decision trees with a maximum depth of 3 and 50 features gave the best result (Fig. 4/b).

k-fold (k = 10) cross validation was run with the best hyperparameters (Fig. 4/c).

In every split, AUROC was calculated which resulted in a mean AUROC of 0.95 with a +- 0.01 standard deviation on the test dataset. The accuracy of the random forest classifier was 0.878 in the test set. The model correctly predicted 83% and 89% of NUMTs (17 804 correctly predicted instances) and random sequences (20 133 correctly predicted instances) of the test set respectively.

NMBroto proved to be the most important feature, while NAC and Z-curve features were the least important features in the classification of NUMTs and random sequences of the test set. Besides NMBroto, Kmer type1, RCKmer type1 and mismatch profile features were also important (Fig. 4/d).

### Machine learning approach for flanking sequence classification

**Fig. 5:**
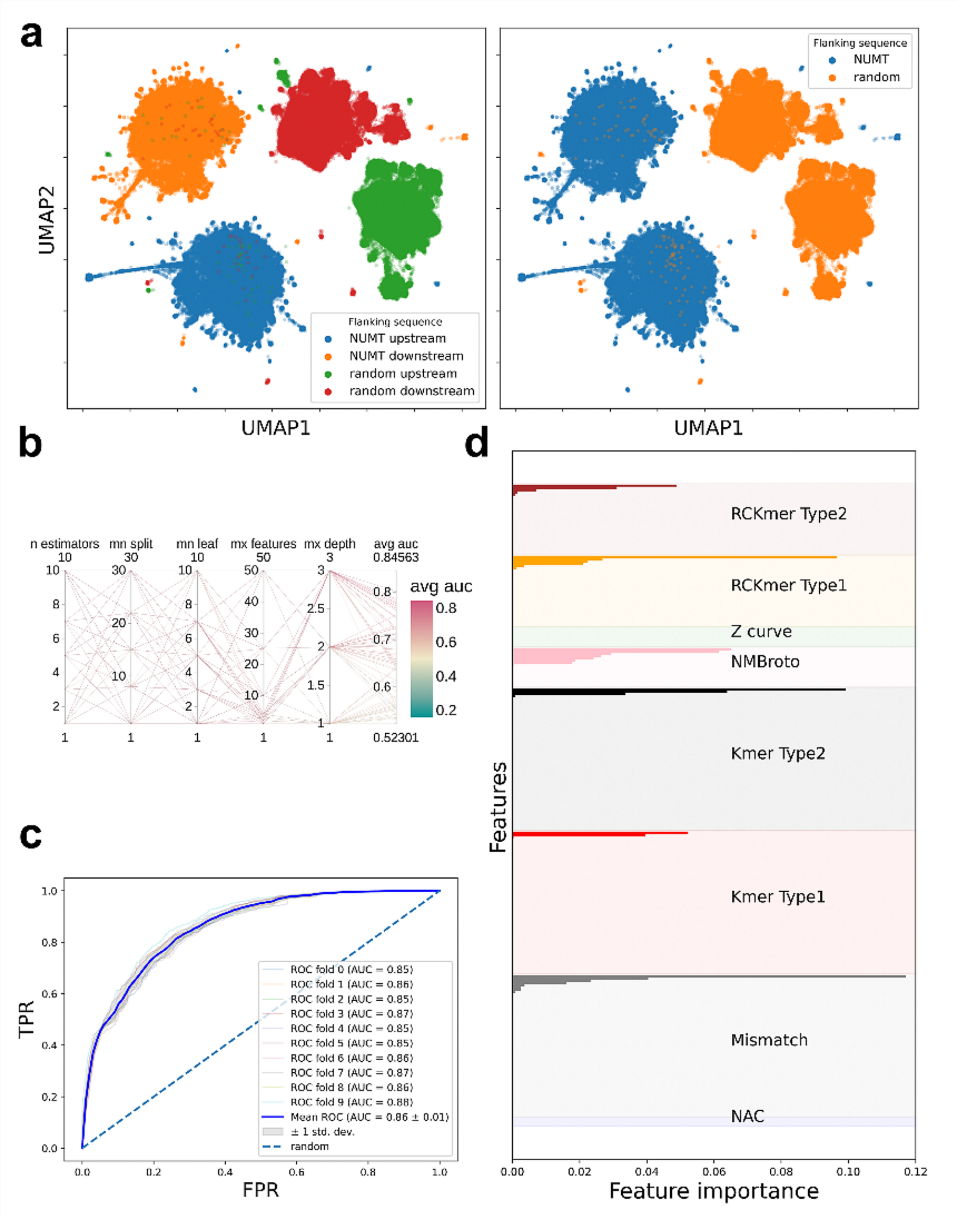
NUMT and random sequence flanking classification with random forest algorithm. (**a**) UMAP dimension reduction of NUMT and random flanking sequence features. (**b**) k-fold cross validation ROC analysis. (**c**) Parallel coordinates diagram of the hyperparameter optimisation. (**d**) Feature importance of iFeatureOmegaCLI’s features

Surprisingly, the flanking regions of NUMTs and random sequences also form separated UMAP clusters. On top of that, using UMAP, the flanking positions (whether it is an upstream or downstream flanking of a random sequence etc.) can also be separated. However, some small upstream flanking clusters of random sequences are closer to the clusters of downstream flanking of random sequences (Fig. 5/a).

In the case of flanking sequence classification, the random forest algorithm’s performance was worse than the observation made during the classification of NUMTs and random sequences. However, the performance is still good, since the random search hyperparameter optimisation resulted in a maximum AUROC of 0.85 (Fig. 5/b).

Throughout the classification, 7 decision trees with a maximum depth of 3 and 50 features gave the best result (Fig. 5/b).

During k-fold (k = 10) cross validation, the mean AUROC was 0.86 with +- 0.01 standard deviation on the test dataset (Fig. 5/c).

The accuracy of the random forest classifier was about 0.774 in the test set. The model correctly predicted 68% and 73% of NUMTs’ flankings (29 374 correctly predicted instances) and random sequences’ flankings (30 373 correctly predicted instances) of the test set respectively.

It turned out that NAC and Z-curve features do not contribute to the classification, hence these two features had 0.0 feature importance. However, there is no such positive outlier importance of a particular feature as we saw in the case of NUMT and random sequence classification (Fig. 5/d).

## Discussion

We observed repetitive elements that have altered presence next to NUMTs (Fig. 1).

The uneven distribution of certain repetitive elements in the close proximity of NUMTs have already been explored in several mammalian species. For example, it has been reported that in the case of several bat nuclear DNAs, the frequency of repetitive elements around NUMTs differ (higher density in the proximity of NUMT, lower density in the proximity of NUMT or no patterns at all) from other ‘non-NUMT’ regions of the genome [31]. In that study the authors came up with a theory for the co-occurrence of NUMTs and repetitive elements. Their explanation is that the repetitive elements that are enriched next to NUMTs are mainly mobile DNA related elements and so those elements can be responsible for NUMT propagation due to a copy-paste mechanism [31]. In the bovine genome, SINE and LINE elements proved to be the most frequent repetitive elements in NUMT regions and in NUMTs themselves too. These repetitive element integration events can contribute to NUMT evolution [32]. SINEs and simple repeats are also enriched in the NUMTs of the human genome [33]. However, based on this study, repetitive element integrations into NUMTs are extremely rare events (0.1% of the 66 000 genomes investigated) which mainly occur in tumour specific NUMTs. And so, the germline transmission of repetitive elements containing NUMTs is highly unlikely hence the repetitive elements cannot contribute to the evolution of NUMTs. Our result show that it is quite common that repetitive elements have their corresponding highest frequencies inside NUMTs. However, we did not investigate the tumour-specificity of these NUMTs. From these repetitive elements SINE MIR, SINE Alu and SINE B4 are non-autonomous, Class I transposable elements (TEs). One of their structural characteristics is a recognition site for a LINE mediated retrotransposition [34, 35]. As to our current knowledge, there is no evidence of SINE mediated NUMTogenesis. However, SINE elements (especially Alu) were reported as contributors to mitochondrial ROS generation and transition pore opening during ageing which are prerequisites of NUMT integration [36, 37]. Furthermore, Alu elements are frequently enriched within genes that are associated with mitochondrial transport processes [38] which supports our findings (Fig. 1).

From the LINE repetitive element class, LINE L1 repetitive element has its peak frequency in NUMTs. LINE L1 is the only autonomously active retrotransposon in the human genome. The components of LINE L1 code for open reading frame 1 and 2 proteins (ORF1p and ORF2p respectively). ORF1p is associated with the L1 RNA to form a chaperon, while ORF2p has retrotransposon related endonuclease and reverse transcriptase activities [39]. Mitochondrial inner and outer membrane translocases TIMM13 and TOMM40 do interact with ORF2p, while the mitochondrial GTPase interacts with ORF1p [40]. Additionally, it has also been described that at DSB sites, LINE L1 retrotransposition occurs more frequently [39] just as it has also been previously proved in case of NUMTs [41]. LTRs are retrotransposons that contain protein coding regions in between two long terminal repeat ends. One of their superfamilies is the superfamily of endogenous retroviruses (ERV) [42]. ERV derived transcripts are strongly connected to highly complex processes which can facilitate mitochondrial membrane permeabilization, hence can facilitate the escape of mitochondrial content into the cytosol [43]. Furthermore, cancerous human cells tend to accumulate ERV transcripts [44].

Based on our results, there is a moderate positive correlation between the size of the nuclear genome and number/total length of NUMTs (Fig. 2/a).

This means that to some extent, the bigger the nuclear genome, the more NUMTs are integrated. The same pattern was found in the case of eukaryotic genomes [27, 45]. This phenomenon is possibly due to the elevated number of DSBs in bigger genomes [27]. However, this motif is non general since in some scenarios these two features (genome size and NUMTs) are just weakly correlated [29], or not correlated at all [46]. We found a modest negative correlation between total nuclear genome GC content and number/total length of NUMTs (Fig. 2/b).

This indicates that the smaller the GC content of a genome, the more NUMTs are integrated into it. However, on the other hand, it is worth noting that the previously described results regarding the correlations were conducted on different taxonomical groups with different methods. It has been previously reported, that during certain types of cancerous transformation, NUMTs tend to integrate into gene rich regions with elevated GC contents [21]. This means that there is going to be a negative selection against NUMT integrations into genomic parts with high GC contents since those NUMTs can disrupt gene functions and cause cancer [33]. The sizes of NUMTs proved to be skewed towards shorter sequences with very few longer ones (Fig. 2/d).

Our results are consistent with the ones published before regarding the human genome when it comes to size distribution of NUMTs [33]. However, megaNUMTs, which we have described in the cases of *Delphinapterus leucas, Tursiops truncatus, Castor canadensis, Cavia porcellus, Globicephala melas, Lagenorhynchus obliquidens, Monodelphis domestica, Orcinus orca, Theropithecus gelada* and *Tursiops truncatus* have been already reported beforehand [7, 8, 47].

NUMTs form order specific clusters based on the extracted features. The taxonomically characteristic patterns of NUMTs make these sequences applicable in phylogenetic studies [48–50] (Fig. 3/a).

Despite the taxonomically well-defined inner structure of NUMT sequences, the process of NUMTogenesis itself seems to be largely universal across the species that were investigated in this study. Meaning that NUMTs are from the whole mtDNA genome, however local ‘hotspots’ (subsequences that contributed to NUMTogenesis more frequently) do exist (Fig. 3/c). Interestingly, we found out that from the dataset investigated in this study, *Primates* was the only order that displayed a well-defined cluster when it came to nucleotide involvement in NUMTogenesis. A possible explanation to this phenomenon is the conserved regions of the mtDNA genomes across *Primates* [51–53]. However, mtDNA is considered to be conserved, environmental factors seem to be highly influential to the structure and plasticity of it. For example, energy need and behaviour have high impact on the selective exposure of different parts of mtDNA [54]. Hence, mammals with bigger brain sizes tend to have different mtDNA compared to others. This difference is due to altered nucleotide substitution rates that makes catalytic activity modifiable through mitochondrial coded enzymes [55]. In our dataset, diverse spectra of mammals were included. In this case, the word ‘diverse’ can be interpreted on many levels such as body composition, energy need or even habitat [56]. As a speculative explanation, this diversity could be considered as a causing factor for the heterogeneities through the organisms investigated here.

NUMTs and random sequences form separated UMAP clusters (Fig. 4/a).

The observation is not surprising considering the fact that NUMTs are from the mtDNA genome which operates with a different genetic code compared to the nuclear genome [57]. The random forest classifier effectively distinguishes between NUMTs and random sequences, resulting in a 0.95 mean AUROC value during k-fold cross validation on the test set (Fig. 4/a, b, c).

NMBroto features had the highest feature importances, i.e. NMBroto contributed to the classification to the largest extent (Fig. 4/d).

This feature is a kind of a normalized autocorrelation measure, which symbolizes the statistical pattern of a biological sequence [58]. In other words, this feature tells us whether a property of a given nucleotide is independent from the same property of the neighbouring nucleotides [59]. NAC and Z-curve features do not contribute to the classification of NUMTs and random sequences. Both features reflect the composition of a sequence, which makes this result more interesting considering the previously mentioned fact that nuclear and mitochondrial DNA (hence NUMTs also) use different genetic codes.

The flanking regions of NUMTs and random sequences also formed well distinguished clusters. Even more remarkable is the result of the upstream and downstream flanking regions of NUMTs and random sequences clustered separately (Fig. 5/a).

It is also possible to classify the flanking regions (whether the given flanking region belongs to a NUMT or a random sequence) using a random forest-based machine learning approach (Fig. 4/c).

There is no such positive outlier in the feature importances as we have previously seen in the case of NUMT and random sequence classification. Moreover, NAC and Z-curve features did not contribute to the classification in this task either (Fig. 4/d).

It is worth mentioning that the NUMT classification algorithm that we have used in this experiment has its drawbacks. This can be seen when it comes to flanking region classification (considering the lower AUROC values). However, the random forest algorithm is still considered a reliable and widely used method in biological studies [60–63], mainly due it’s results interpretability and “non-black-box” nature.

## Conclusions

In this article we characterised the NUMTs of all the available mammalian genomes at the NCBI database for the first time as to our current knowledge. We described several repetitive elements that show altered presence in the proximity of NUMTs. With the use of different features of nucleic acid sequences, we were able to classify NUMTs and also their corresponding flanking regions. Our results on the large dataset of mammalian species contribute to the theory that NUMT insertion is non-random.

To achieve our goal, we used machine learning methods that have not yet appeared in the NUMT literature hence can be considered as cutting-edge technology. Due to their ability to analyse large amounts of biological data and make accurate decisions and predictions without being explicitly programmed, machine learning methods become increasingly popular in bioinformatics. With machine learning methods, one can identify patterns and relationships in complex and high-dimensional data that may be missed with traditional statistical methods. Furthermore, NUMT prediction also has a practical benefit since NUMT integration can lead to bias during molecular works or even non accurate genome assembly. With the help of our predictive model, NUMTs can be accurately identified to eliminate their impact on the downstream analyses. Another important result of our experiments is that we have built and published here a platform that can be used by anyone, with the help of which cross-species NUMT characterization projects will finally become comparable and more manageable. This workflow can be used easily and quickly on large data, which greatly facilitates the mining of NUMTs in all genomes.

Integration of NUMTs into the nuclear genome de novo may play important roles in the development of various diseases and ageing. The increased NUMT integration with elevated temperature or increased ROS exposure, raises the question of how the amount of NUMTs in genomes will change due to climate change and the rising toxin levels found in food. A thorough understanding of the process of NUMTogenesis would give us the potential to provide greater insight into the biological relevance of the role of NUMTs in ageing, cancer, and genome integrity. By elucidating the exact mechanisms underlying NUMTogenesis, we can better understand and interpret the role of NUMTs in the genomes. This study provides a valuable contribution in two aspects. Firstly, it presents an analysis conducted on mammalian species, shedding light on the NUMTogenesis processes occurring in their genomes. Secondly, it introduces a novel workflow that facilitates the comparative analysis of these processes across diverse genomes.

## Methods

### Genome curation

A total number of 153 nuclear and mtDNA genomes were analysed. All the reference nuclear and mtDNA genome sequences were acquired from the FTP site of NCBI. Taxonomical related data is also from NCBI data source. The exact URLs are in the Availability of data and materials section.

### Sequence alignments

The sequence alignment of the corresponding genomes was performed using LASTAL (v.: v1219) [64] with the settings and e value threshold as it has been described beforehand [65]. We chose LASTAL since it is believed to provide more accurate results for e value calculations than other widely used methods [65]. The sequence alignments of the 153 genomes resulted in 79 645 NUMTs.

While the multiple sequence alignment of the mitochondrial sequences was performed with ClustalO (v.: 1.2.4) [66] with default parameters.

Repetitive elements were investigated with RepeatMasker (v.: 4.1.2.p1) [67] using species specific settings.

### Feature extraction

Nucleic acid composition (NAC), kmers (Kmer type 1 and 2, RCKmer type 1 and 2), normalized Moreau-Broto autocorrelation (NMBroto), Z-curve (geometrical features of a nucleic acid sequence [68]) and mismatch profile features were extracted with iFeatureOmegaCLI (v.: 1.0.2) [58] for further classification. IFeatureOmegaCLI provides a high-throughput, robust, easy to automate workflow for extracting meaningful features from biological sequences.

### Machine learning

Uniform Manifold Approximation and Projection (UMAP), classification, grid search, random search and k-fold (k = 10) cross validation algorithms were implemented in Scikit-learn (v.: 1.0.2) [69]. During grid and random search, the corresponding hyper parameters were optimised. The best hyper parameter combination was selected based on the area under receiver operating characteristic curve (AUROC score). We decided to use the Scikit-learn implementation of the above-mentioned algorithms since these models are scalable and the resulting models are easy to share cross platforms.

### Negative labelled sequence identification

For negative labelled samples acquisition, we generated as many random positions for a given genomic id as many NUMTs were located on the given genomic part. In this way we downsampled the majority class (“negative” sample sequences at random positions). This resulted in the exact same number of random sequences as many NUMTs we explored for the sake of a balanced dataset for the classification. Then, a sequence was extracted using Samtools (v.: 1.6) [70] starting from the previously determined random position in a length of the corresponding NUMTs that were integrated into the same genomic part. For instance, if the X chromosome of the mouse genome contains 2 NUMTs in a length of 123 and 304 bps, then this chromosome is going to be sampled two times at random positions in the same lengths i.e., 123 and 304 bps, respectively. As we have pointed out previously, this sampling technique provides a totally balanced dataset which is crucial when it comes to training ML models without overfitting or other biased behaviour.

### Phylogenetic tree construction

Ape (v.: 5.6-2) [71] and phangorn (v.: 2.10.0) [72] libraries were used during phylogenetic analysis. The consensus phylogenetic tree was constructed from a bootstrap dataset with 100 bootstrap iterations using the GTR nucleic acid substitution model for optimizing the JC69 model.

### General statistics

The statistical analysis (Spearman correlation, Kolmogorov-Smirnov test) was carried out in Scipy (v.: 1.7.3) [73]. Statistics with *p* value < 0.05 were considered as significant results.

Relative GC contents were calculated using whole genome GC contents derived from NCBI. The exact URL is in the Availability of data and materials section.

The NUMT mining application and the trained model are available at: https://github.com/balintbiro/NUMT_finder.

**Fig. 6:**
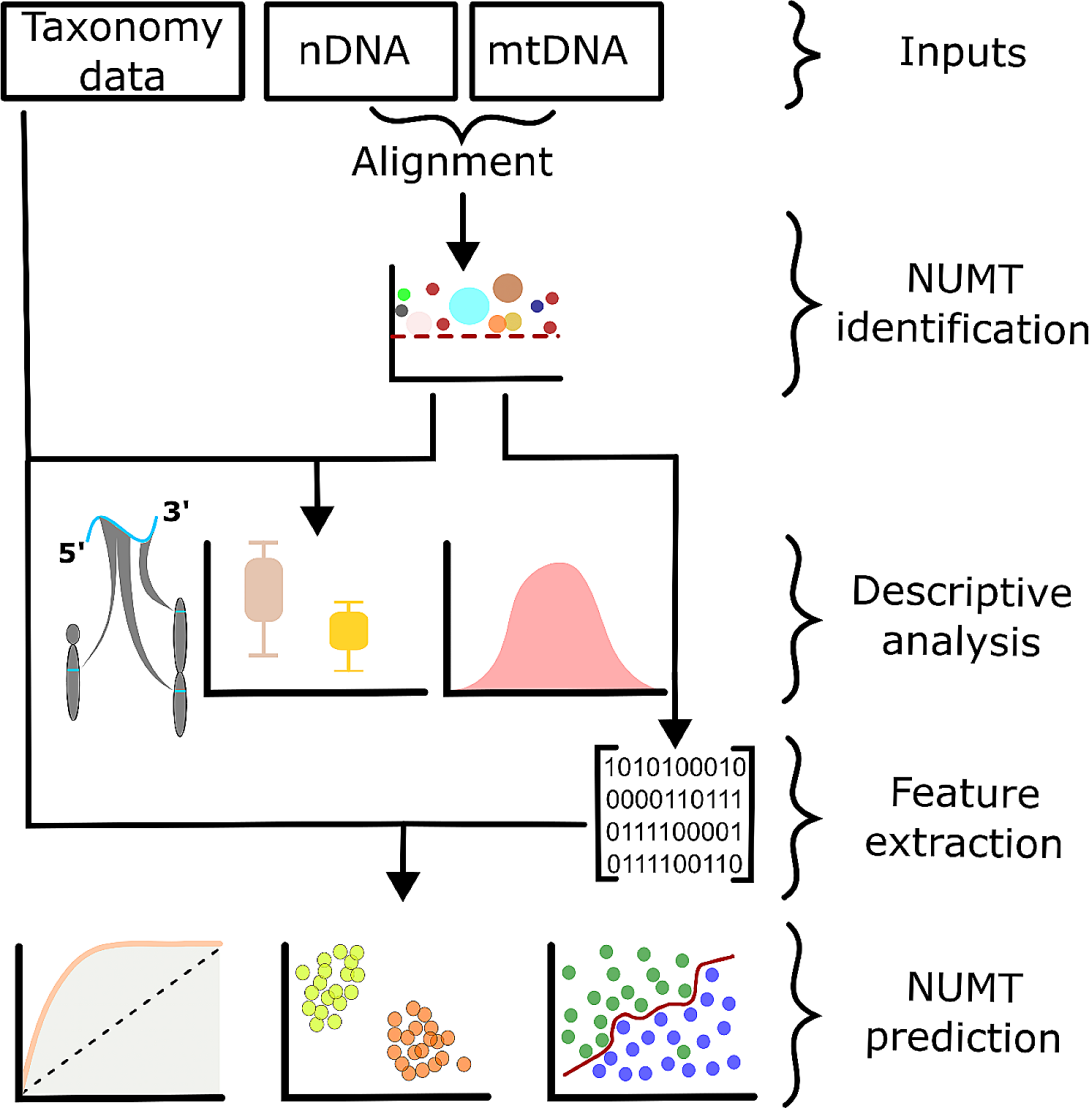
Graphical summary of the methods

The workflow of our experiments can be seen on Fig. 6.

## Electronic supplementary material

Below is the link to the electronic supplementary material.

Supplementary Material 1

## Data Availability

All the nuclear and mtDNA genome sequences are available on the FTP site of NCBI (https://ftp.ncbi.nlm.nih.gov/genomes/refseq/vertebrate_mammalian/; https://ftp.ncbi.nlm.nih.gov/genomes/refseq/mitochondrion/). Taxonomical related data is also from NCBI data source (https://ftp.ncbi.nlm.nih.gov/genomes/GENOME_REPORTS/eukaryotes.txt). The NUMT mining application and custom BASH, R and Python codes are available at GitHub repositories (https://github.com/balintbiro/NUMT_finder; https://github.com/balintbiro/numt_mining). Supplementary Information contains a file with the species names involved in the analysis.

## Abbreviations

AUROC: Area Under the Receiver Operating characteristic Curve
DNA hAT-Charlie: hAT-Charlie DNA transposon
DSB: Double Stranded DNA Break
EGT: Endosymbiotic Gene Transfer
ERVL: Endogenous RetroVirus-related eLement
GC: Guanine-Cytosine ratio
GTR: General Time Reversal nucleic acid substitution model
nDNA: Nuclear genomic DNA
LINE: Long Interspersed Nuclear Element
LTR: Long Terminal Repeat
Mb: Mega base
NCBI: National Centre for Biotechnology Information
NAC: Nucleic Acid Composition
NHEJ: Non-Homologous End Joining DNA repair mechanism
NUMT: Nuclear Mitochondrial Sequence
NUMTogenesis: The process in which NUMTs are created
mtDNA: Mitochondrial DNA
ROS: Reactive Oxygen Species
SINE: Short Interspersed Nuclear Element
TRNA: Transfer RNA
UMAP: Uniform Manifold Approximation and Projection for dimension reduction
